# Supplementary material for: Climate resilience through bioeconomy: A mixed-methods protocol for assessing adaptation policies in rural settlements on the Amazon
Source: PLoS One. 2026 Feb 13;21(2):e0342911. doi: 10.1371/journal.pone.0342911 (PMC12904387; doi:10.1371/journal.pone.0342911)
Supplement: S1 File — (PDF) [file pone.0342911.s001.pdf]

HOUSEHOLD QUESTIONNAIRE

SECTION 1: SOCIOECONOMIC AND DEMOGRAPHIC CHARACTERISTICS

1.1. Personal Data
Full Name: ________________________________
Sex: ( ) Male   ( ) Female   ( ) Prefer not to say
Date of Birth: __/__/__
Place of Birth: ____________________

1.2. Race/Ethnicity
( ) White
( ) Black
( ) Mixed-race (Pardo)
( ) Indigenous
( ) Other

1.3. Marital Status:
( ) Single
( ) Married or in a stable union
( ) Separated/Divorced
( ) Widowed

1.4. Education of the Household Head:
( ) No formal education
( ) Incomplete primary education
( ) Complete primary education
( ) Incomplete secondary education
( ) Complete secondary education
( ) Incomplete higher education
( ) Complete higher education

1.5. Household Composition
Girls (under 14 years old): _________
Women (14 years old and over): _________
Boys (under 14 years old): _________
Men (14 years old and over): _________

1.6. Monthly Household Income
( ) Less than 1 Minimum Wage
( ) 1 – 2 Minimum Wages
( ) More than 2 Minimum Wages

1.7. Does the household receive any social program benefits? (check all that apply)
( ) Bolsa Família / Auxílio Brasil
( ) BPC (Continuous Cash Benefit)

( ) Rural Retirement Pension
( ) Others: __________________
( ) None

1.8. When did you move to this settlement?
Year: ___________

1.9. Has the household lived in another settlement before?
( ) Yes ( ) No
If yes, which one? _____________________

1.10. Did the household move here for reasons related to:
(multiple answers possible)
( ) Access to land
( ) Land conflicts
( ) Search for work
( ) Environmental problems in the previous location (e.g., drought, flooding)
( ) Family reunification
( ) Other: _______________

Section 1.1. Migration

1.1.1. Have you or any member of your household ever migrated temporarily to work elsewhere?
( ) Yes ( ) No

1.1.2. If yes, who migrated?
( ) Household head
( ) Son/Daughter
( ) Spouse
( ) Other: ________
To which city/state? ___________________________

1.1.3. Reason for migration:
( ) Economic (employment, land)
( ) Environmental (drought, flooding, soil degradation)
( ) Land conflict/violence
( ) Family reunification
( ) Other: ____________________

1.1.4. Type of migration:
( ) Temporary (returns to the settlement)
( ) Permanent
( ) Seasonal (during harvests or drought)

1.1.5. How many times have you migrated in the last 10 years?
( ) 1
( ) 2–3
( ) More than 3

1.1.6. Do you consider that your family might migrate in the next 5 years?
( ) Yes, definitely
( ) Maybe
( ) Do not intend to migrate

If yes, probable reason:
( ) Lack of income
( ) Climate conditions
( ) Lack of public policies
( ) Search for study/work opportunities
( ) Other: ________________

SECTION 2: LIVELIHOODS AND WORK

2.1. Main source of household income:
( ) Subsistence farming
( ) Commercial farming
( ) Livestock raising
( ) Social benefits (pension, Bolsa Família)
( ) Work outside the plot (day laborer, public servant, etc.)
( ) Other: __________________
( ) No response

2.2. We have questions about the planting area. How would you prefer to answer?
( ) Hectares (ha)
( ) Rows ( )
( ) Other: ______

2.3. Does the household maintain:
( ) Traditional fields (_____)
( ) Agroforestry systems (_____)
( ) Livestock (_____)
( ) Extractivism (_____)
( ) Others (_____)
( ) No response

2.4. How much time do you spend on these activities?
( ) Up to 8 hours
( ) More than 8 hours

( ) Up to 4 hours
( ) No response

2.5. Household income comes from work that is:
( ) Exclusively local
( ) Predominantly local (1–3 months per year outside)
( ) Seasonal (4–8 months outside)
( ) Predominantly outside (migratory)
( ) No response

2.6. Agricultural production is intended for:
( ) Household consumption only
( ) Sale only
( ) Both consumption and sale
( ) No response

2.7. How are products commercialized?
( ) Direct to consumer
( ) Through cooperatives
( ) To intermediaries
( ) Local markets/fairs
( ) Public policies (e.g., PAA)
( ) Not commercialized
( ) No response

2.8. Has the household participated in training/technical capacity-building in the last 5 years?
( ) Yes
( ) No

If yes, what was the main topic?
( ) Agroecological production
( ) Irrigation techniques
( ) Rural property management
( ) Commercialization/market
( ) Climate adaptation
( ) Forest management
( ) Socio-environmental certifications
( ) Other: _______________
( ) No response

2.9. Is the household registered in *CadÚnico*?
( ) Yes
( ) No
( ) No response

SECTION 3: CHARACTERISTICS OF THE AGRICULTURAL ESTABLISHMENT

3.1. How do you prefer to measure your area?
( ) Hectares
( ) Rows
( ) *Alqueires* (local unit)
( ) Other: ______

3.2. Total area of the plot: _____

3.3. Area used for:

- Temporary crops: ______ ha
- Permanent crops: ______ ha
- Planted pasture: ______ ha
- Native pasture: ______ ha
- Agroforestry / SAFs: ______ ha
- Preserved native vegetation: ______ ha
- Secondary vegetation (*capoeira*): ____ ha
- Permanent preservation area (APP): ___ ha
- Other uses: ______________________ ha

3.4. Does the household raise animals?
( ) Yes
( ) No
( ) No response

If yes, please fill in the table:

Type of animal	Number of animals	Production (liters, dozens, etc.)	Destination (Consumption, Sale, Both)

3.5. Do you have any irrigation system for agricultural production?
( ) Yes
( ) No
( ) No response

3.6. If yes, which type of irrigation is used?
( ) Sprinkler
( ) Drip irrigation

( ) Furrow/canal irrigation
( ) Other: _______________
( ) No response

3.7. What is the source of water used for irrigation?
( ) Well
( ) Stream or river
( ) Cistern
( ) Community system
( ) Other: ___________________
( ) No response

3.8. Do you have a cistern for rainwater collection/storage?
( ) Yes
( ) No
( ) No response

3.9. In the past 10 years, have you experienced water shortages for PRODUCTION?
( ) Yes
( ) No
( ) No response

3.10. If yes, what was the maximum period you suffered without water or with insufficient availability?
( ) 1–3 months
( ) 4–6 months
( ) More than 6 months
( ) No response

3.11. How do you rate the change in water availability for PRODUCTION?
(1 = not serious / 5 = very serious)
( ) 1 ( ) 2 ( ) 3 ( ) 4 ( ) 5

3.12. Have you noticed changes in water availability for CONSUMPTION in the past 10 years?
( ) Yes
( ) No
( ) No response

3.13. If yes, what was the maximum period you suffered without water or with insufficient availability for CONSUMPTION?
( ) 1–3 months
( ) 4–6 months
( ) More than 6 months
( ) No response

3.14. How do you rate the change in water availability for CONSUMPTION?
(1 = not serious / 5 = very serious)
( ) 1 ( ) 2 ( ) 3 ( ) 4 ( ) 5

3.15. Does the household use pesticides or chemical fertilizers in production?
( ) Yes
( ) No
( ) Only organic inputs
( ) No response

3.16. Is the household's production certified or part of traceability initiatives?
( ) Yes, has organic certification
( ) Yes, participates in traceability
( ) No certification
( ) No response

3.17. Does the household share the use of the plot with other family units (e.g., married children, relatives)?
( ) Yes
( ) No
( ) No response

3.18. Has the household obtained any financing or loans in the past 5 years?
( ) Yes
( ) No
( ) No response

3.19. What was the purpose?
( ) Investment
( ) Operating costs
( ) Commercialization
( ) Maintenance
( ) No response

3.20. From whom did you receive it?
( ) Banks
( ) Cooperative
( ) Government programs
( ) Relatives or friends
( ) Others
( ) No response

SECTION 4: DEPENDENCE ON NATURAL RESOURCES

4.1. Does the household use natural resources from the forest?
( ) Yes

( ) No
( ) No response

If yes, which ones?
( ) Timber
( ) Brazil nuts
( ) Açaí
( ) Honey
( ) Babassu
( ) Medicinal plants
( ) Others: ____________

4.2. Frequency of collection:
( ) Daily
( ) Weekly
( ) Monthly
( ) Seasonal
( ) No response

4.3. Main purpose:
( ) Subsistence
( ) Local trade
( ) Formal value chains (companies, government, others)
( ) No response

4.4. How is this resource obtained?
( ) Simple gathering
( ) Sustainable management
( ) Agroforestry systems
( ) No response

4.5. Has the household noticed changes in the availability of natural resources (timber, fruits, etc.) in the past 10 years?
( ) Yes
( ) No
( ) Don't know

If yes, specify: _____________________________________

4.6. Has the household faced any of the following situations:
( ) Conflicts over access to resources
( ) Conflicts over collection/extractive areas
( ) Pressure from external deforestation
( ) None

SECTION 5: PERCEPTIONS OF CLIMATE CHANGE

5.1. Have you ever heard about "climate change"?

( ) Yes
( ) No
( ) No response

5.2. Where did you obtain this information?

( ) Radio
( ) TV
( ) Technical agents
( ) Organizations
( ) Social media
( ) Other, specify: ___________________________
( ) No response

5.3. On a scale of 1 to 5, how much do you perceive that the climate has changed in the past 10 years?

(1 = Did not perceive any changes | 5 = Perceived many changes)
( ) 1 ( ) 2 ( ) 3 ( ) 4 ( ) 5 ( ) No response

5.4. Which climate changes have you noticed? (check all that apply)

( ) Increase in average temperature
( ) Reduction in rainfall
( ) Delay or irregularity in the onset of rains
( ) Longer dry periods
( ) Heavier rainfall in shorter periods
( ) More intense frosts or cold spells
( ) Floods or inundations
( ) Changes in wind patterns
( ) Alterations in river/stream flow
( ) Changes in fruiting periods
( ) Emergence of new pests
( ) Others: ____________________
( ) No response

5.5. In which periods of the year are these climates changes noticed?

( ) Winter
( ) Summer
( ) Both
( ) Cannot identify
( ) No response

5.6. In your opinion, what are the causes of these climate changes?

( ) Deforestation
( ) Burning of vegetation
( ) Use of pesticides
( ) Industrial activities

( ) Urban expansion
( ) Mining activities
( ) Natural causes
( ) Others: ____________________
( ) Don’t know
( ) No response

5.7. On a scale of 1 to 5, how concerned are you about climate change?
(1 = Not concerned at all | 5 = Very concerned)
( ) 1 ( ) 2 ( ) 3 ( ) 4 ( ) 5 ( ) No response

5.8. Do you consider climate change to be a direct threat to your family or your production?
(1 = Strongly disagree | 5 = Strongly agree)
( ) 1 ( ) 2 ( ) 3 ( ) 4 ( ) 5 ( ) No response

5.9. Have climate changes directly affected the following? (check all that apply)
( ) Agricultural calendar
( ) Crop productivity
( ) Animal health
( ) Family members’ health
( ) Storage conditions
( ) Household income
( ) Infrastructure (roads, energy, etc.)
( ) Water quality
( ) Food security
( ) Availability of forest products
( ) No response

5.10. Do you consider that you have sufficient access to information to understand what is happening with the climate?
( ) Yes
( ) No
( ) Partially
( ) No response

SECTION 6: VULNERABILITIES

6.1. Has your production been affected by extreme climate events in the past 5 years?
( ) Yes
( ) No
( ) No response

If yes, indicate the type of event and the year(s):

Climate event	Year(s) occurred	Impact on production (check)
Prolonged drought		
Heavy rains		
Flash floods/flooding		
Severe cold/frost		
Other: _____________		

6.2. Rate the impact of climate change on:
(1 = None | 5 = Severe)

Activity/Resource 1 2 3 4 5

Main agriculture

Livestock

Extractivism

Water availability

Family health

6.3. Has the household experienced income losses due to these events?
( ) Yes
( ) No
( ) No response

6.4. Has the household received any public or private support to cope with these losses?
( ) Yes
( ) No
( ) No response

If yes, which ones?
( ) Emergency credit
( ) Distribution of food/inputs
( ) Technical assistance
( ) Others: ______________________
( ) No response

6.5. Which strategies has the household used to cope with climate-related losses?
( ) Reduction of production
( ) Taking on debt
( ) Help from relatives or neighbors

( ) Temporary migration for external work
( ) Cutting essential expenses
( ) Sale of animals or assets
( ) No specific strategy
( ) Others: ______________________
( ) No response

6.6. To what extent does your household feel prepared to cope with extreme climate events (e.g., drought, heavy rainfall)?
(1 = Not prepared | 5 = Very prepared)
( ) 1
( ) 2
( ) 3
( ) 4
( ) 5
( ) No response

SECTION 7: ADAPTATION AND MITIGATION ACTIONS

7.1. Do you know what climate adaptation and mitigation are?
( ) No
( ) Yes
( ) I have heard about it, but I don't know exactly what it is
( ) No response

7.2. Has the household adopted any of the practices below to adapt to climate change?
( ) Yes
( ) No
( ) No response

If yes, which ones?
( ) Planting at a different time
( ) Cultivation of more resistant varieties
( ) Use of irrigation
( ) Agroforestry systems
( ) Crop rotation
( ) Others: ______________________
( ) No response

7.3. Has the household participated in any public policy programs in the past 5 years? (check all that apply)

Policy	Accessed?	Faced difficulties?	Consider it useful for addressing climate change?
PRONAF	( ) Yes / ( ) No	( ) Yes / ( ) No	( ) Yes / ( ) No
PAA	( ) Yes / ( ) No	( ) Yes / ( ) No	( ) Yes / ( ) No
PNAE	( ) Yes / ( ) No	( ) Yes / ( ) No	( ) Yes / ( ) No
INCRA support program	( ) Yes / ( ) No	( ) Yes / ( ) No	( ) Yes / ( ) No
Agricultural insurance / Crop guarantee	( ) Yes / ( ) No	( ) Yes / ( ) No	( ) Yes / ( ) No
Technical assistance (public or private ATER)	( ) Yes / ( ) No	( ) Yes / ( ) No	( ) Yes / ( ) No
Environmental regularization program (CAR, PRA)	( ) Yes / ( ) No	( ) Yes / ( ) No	( ) Yes / ( ) No
NGO/organization projects	( ) Yes / ( ) No	( ) Yes / ( ) No	( ) Yes / ( ) No

7.4. Have you received guidance on how to adapt to climate change from:
( ) Cooperative technician
( ) EMATER or public agency
( ) NGO
( ) University or research institute
( ) Other: ___________________
( ) Never received
( ) No response

7.5. To what extent do you trust that current public policies adequately support farmers in facing climate change?
(1 = No trust | 5 = A lot of trust)
( ) 1
( ) 2
( ) 3
( ) 4
( ) 5
( ) No response

7.6. Would you like to receive more information on how to adapt your production to climate change?
(1 = Not interested | 5 = Very interested)
( ) 1

( ) 2
( ) 3
( ) 4
( ) 5
( ) No response

7.7. Is migration a way to reduce climate risks?
(1 = Strongly disagree | 5 = Strongly agree)
( ) 1
( ) 2
( ) 3
( ) 4
( ) 5
( ) No response

7.8. Do you know or have you heard about "agroecology," "agroecological transition," or "sustainable production"?
( ) Yes
( ) No
( ) Have heard of it, but don't know well
( ) No response

7.9. Does the household adopt any of the practices below?
( ) Pasture recovery
( ) Crop-livestock-forest integration (ILPF)
( ) Composting
( ) Sustainable vegetation management
( ) No-till farming
( ) None
( ) No response

SECTION 8: BIOECONOMY

8.1. Have you ever heard about Bioeconomy?
( ) Yes
( ) No
( ) No response

8.2. Do you produce any of the products below?

**Product**	**Production Syste**	**Approx. Annual Volume**	**Is it processed?**	**Certification**	**Importance for Household Income**
( ) Açaí	( ) Extractivism<br>( ) Cultivation<br>( ) Mixed		( ) Yes<br>( ) No	( ) Organic<br>( ) Socio-biodiversity<br>( ) Other<br>( ) None	( ) No income<br>( ) Small income<br>( ) Medium income<br>( ) Main income

Product	Production Syste	Approx. Annual Volume	Is it processed?	Certification	Importance for Household Income
( ) Brazil Nut	( ) Extractivism<br>( ) Cultivation<br>( ) Mixed		( ) Yes<br>( ) No	( ) Organic<br>( ) Socio-biodiversity<br>( ) Other<br>( ) None	( ) No income<br>( ) Small income<br>( ) Medium income<br>( ) Main income
( ) Andiroba	( ) Extractivism<br>( ) Cultivation<br>( ) Mixed		( ) Yes<br>( ) No	( ) Organic<br>( ) Socio-biodiversity<br>( ) Other<br>( ) None	( ) No income<br>( ) Small income<br>( ) Medium income<br>( ) Main income
( ) Cacao	( ) Extractivism<br>( ) Cultivation<br>( ) Mixed		( ) Yes<br>( ) No	( ) Organic<br>( ) Socio-biodiversity<br>( ) Other<br>( ) None	( ) No income<br>( ) Small income<br>( ) Medium income<br>( ) Main income
( ) Honey	( ) Extractivism<br>( ) Cultivation<br>( ) Mixed		( ) Yes<br>( ) No	( ) Organic<br>( ) Socio-biodiversity<br>( ) Other<br>( ) None	( ) No income<br>( ) Small income<br>( ) Medium income<br>( ) Main income
( ) Peach Palm	( ) Extractivism<br>( ) Cultivation<br>( ) Mixed		( ) Yes<br>( ) No	( ) Organic<br>( ) Socio-biodiversity<br>( ) Other<br>( ) None	( ) No income<br>( ) Small income<br>( ) Medium income<br>( ) Main income
( ) Babassu	( ) Extractivism<br>( ) Cultivation<br>( ) Mixed		( ) Yes<br>( ) No	( ) Organic<br>( ) Socio-biodiversity<br>( ) Other<br>( ) None	( ) No income<br>( ) Small income<br>( ) Medium income<br>( ) Main income
( ) Anatto (Urucum)	( ) Extractivism<br>( ) Cultivation<br>( ) Mixed		( ) Yes<br>( ) No	( ) Organic<br>( ) Socio-biodiversity<br>( ) Other<br>( ) None	( ) No income<br>( ) Small income<br>( ) Medium income<br>( ) Main income
( ) Bacuri	( ) Extractivism<br>( ) Cultivation<br>( ) Mixed		( ) Yes<br>( ) No	( ) Organic<br>( ) Socio-biodiversity<br>( ) Other<br>( ) None	( ) No income<br>( ) Small income<br>( ) Medium income<br>( ) Main income
( ) Cupuaçu	( ) Extractivism<br>( ) Cultivation<br>( ) Mixed		( ) Yes<br>( ) No	( ) Organic<br>( ) Socio-biodiversity<br>( ) Other<br>( ) None	( ) No income<br>( ) Small income<br>( ) Medium income<br>( ) Main income
( ) Rubber	( ) Extractivism<br>( ) Cultivation<br>( ) Mixed		( ) Yes<br>( ) No	( ) Organic<br>( ) Socio-biodiversity<br>( ) Other<br>( ) None	( ) No income<br>( ) Small income<br>( ) Medium income<br>( ) Main income
( ) Buriti Palm	( ) Extractivism<br>( ) Cultivation<br>( ) Mixed		( ) Yes<br>( ) No	( ) Organic<br>( ) Socio-biodiversity<br>( ) Other<br>( ) None	( ) No income<br>( ) Small income<br>( ) Medium income<br>( ) Main income
( ) Copaíba	( ) Extractivism<br>( ) Cultivation<br>( ) Mixed		( ) Yes<br>( ) No	( ) Organic<br>( ) Socio-biodiversity<br>( ) Other<br>( ) None	( ) No income<br>( ) Small income<br>( ) Medium income<br>( ) Main income
( ) Murici	( ) Extractivism<br>( ) Cultivation<br>( ) Mixed		( ) Yes<br>( ) No	( ) Organic<br>( ) Socio-biodiversity<br>( ) Other<br>( ) None	( ) No income<br>( ) Small income<br>( ) Medium income<br>( ) Main income
( ) Tucumã	( ) Extractivism<br>( ) Cultivation<br>( ) Mixed		( ) Yes<br>( ) No	( ) Organic<br>( ) Socio-biodiversity<br>( ) Other	( ) No income

Product	Production Syste	Approx. Annual Volume	Is it processed?	Certification	Importance for Household Income
				( ) None	( ) Small income<br>( ) Medium income<br>( ) Main income
( ) Piquiá	( ) Extractivism<br>( ) Cultivation<br>( ) Mixed		( ) Yes<br>( ) No	( ) Organic<br>( ) Socio-biodiversity<br>( ) Other<br>( ) None	( ) No income<br>( ) Small income<br>( ) Medium income<br>( ) Main income
( ) Cumaru (Tonka Been)	( ) Extractivism<br>( ) Cultivation<br>( ) Mixed		( ) Yes<br>( ) No	( ) Organic<br>( ) Socio-biodiversity<br>( ) Other<br>( ) None	( ) No income<br>( ) Small income<br>( ) Medium income<br>( ) Main income
( ) Bacaba	( ) Extractivism<br>( ) Cultivation<br>( ) Mixed		( ) Yes<br>( ) No	( ) Organic<br>( ) Socio-biodiversity<br>( ) Other<br>( ) None	( ) No income<br>( ) Small income<br>( ) Medium income<br>( ) Main income
( ) Medicinal Plants	( ) Extractivism<br>( ) Cultivation<br>( ) Mixed		( ) Yes<br>( ) No	( ) Organic<br>( ) Socio-biodiversity<br>( ) Other<br>( ) None	( ) No income<br>( ) Small income<br>( ) Medium income<br>( ) Main income
( ) Murumuru	( ) Extractivism<br>( ) Cultivation<br>( ) Mixed		( ) Yes<br>( ) No	( ) Organic<br>( ) Socio-biodiversity<br>( ) Other<br>( ) None	( ) No income<br>( ) Small income<br>( ) Medium income<br>( ) Main income

8.3. What other agricultural products does your household cultivate?

( ) Do not produce any other product
( ) No response

Please fill in the table: ________________________________

8.4. Do you commercialize these products with the support of:

( ) Cooperative
( ) Intermediary
( ) Public program (PAA, PNAE)
( ) Direct sales (markets/fairs, neighbors)
( ) Do not commercialize
( ) No response

8.5. What are the main difficulties in expanding the production of these products in your area?

(multiple answers possible)
( ) Lack of equipment
( ) Lack of technical assistance
( ) Lack of market/buyer
( ) Logistics difficulties
( ) Environmental/health legislation
( ) Lack of infrastructure

( ) Climate impacts
( ) Difficulty with collective organization
( ) Other: ________________
( ) No response

8.6. How does climate change affect these value chains?
( ) Changes in product availability
( ) Changes in production cycles
( ) Storage difficulties
( ) Transportation problems
( ) No perceptible effect
( ) No response

8.7. Is there interest in expanding production and income with these products?
( ) Yes
( ) No
( ) Don't know
( ) No response

8.8. Has the household received public or private support to work with socio-biodiversity products?
( ) Yes
( ) No

If yes, which ones?
( ) Technical training
( ) Equipment
( ) Market access
( ) Specific credit
( ) Others: ______________________
( ) No response

8.9. Does the household participate in a structured value chain for these products?
( ) Yes, through a cooperative or association
( ) Yes, directly with fixed buyers
( ) Does not participate in any structured chain
( ) No response

8.10. Are the young members of the household interested in continuing these activities?
( ) Yes, all
( ) Some
( ) None
( ) Not applicable
( ) No response

8.11. How important are these products for household income?
(1 = Not important | 5 = Very important)
( ) 1
( ) 2
( ) 3
( ) 4
( ) 5
( ) No response

8.12. Would the household be interested in working with new forest products?
( ) Yes. Which? ________________
( ) No
( ) No response
