# Supplementary material for: Climate resilience through bioeconomy: A mixed-methods protocol for assessing adaptation policies in rural settlements on the Amazon
Source: PLoS One. 2026 Feb 13;21(2):e0342911. doi: 10.1371/journal.pone.0342911 (PMC12904387; doi:10.1371/journal.pone.0342911)
Supplement: S2 File — (PDF) [file pone.0342911.s002.pdf]

FOCUS GROUP GUIDE IN SETTLEMENTS

I. Opening and Contextualization

1. Icebreaker Activity:
    - Each participant answers:
        - Name, time living in the settlement.
        - *"One word that describes the climate over the past 5 years"* (e.g., *"unpredictable," "drought"*).
2. Collective Ranking:
    - Rank the following impacts in order of severity (1 to 5):
        - Prolonged drought.
        - Heavy rains/floods.
        - Extreme heat.
        - New pests.
        - Reduction of forest resources.

II. Climate Impacts and Production

Technique: "Spoken Map" (use a map of the settlement to mark critical areas).

1. Key Questions:
    - *"Which areas of the settlement are most affected by climate events? Why?"*
    - *"Has any crop or production system (field crops, pasture, extractivism) become unviable? Which one, and why?"*
    - *"How has access to food from fields or forests changed? Are there fewer fruits, game, or medicinal herbs?"*
2. Quantifiable Data:
    - Collective estimate: *"How many harvests have been lost in the past 3 years due to climate?"*

III. Adaptation Strategies

Technique: Small Group Discussion (divide by gender or productive activity).

1. Key Questions:
    - *“What techniques do you use to reduce risks? (e.g., diversification, water storage, resilient seeds).”*
    - *“Do women and men adopt different strategies? Which ones?”*
    - *“What barriers prevent the adoption of more adaptive practices? (e.g., cost, lack of information).”*
2. Prioritization:
    - List all strategies mentioned and vote on the three most effective.

IV. Community Organization and Gender

Technique: Timeline (climate events × collective responses).

1. Key Questions:

- *“How have household tasks (fetching water, caring for the sick) increased with climate change?”*
- *“Are there initiatives led by women or youth to address these challenges (e.g., seed banks, solidarity funds)?”*
- *“How does the community organize itself to make decisions about natural resources?”*

VI. Value Chains and Markets

Technique: Role-Playing (simulate a dialogue between farmers and buyers).

1. Key Questions:

- *“How have climate events affected commercialization? (e.g., prices, transport, crop losses).”*
- *“Do you participate in formal chains (cooperatives, PAA) or informal ones? Which is more vulnerable to climate impacts?”*
- *“Does anyone here sell products with certification (organic, socio-biodiversity)? How did you obtain it?”*
- *“Does the market pay more for sustainable products? Is it worthwhile?”*

2. Youth and the Future:

- *“Do young people in the settlement see a future in these activities? Why?”*

3. Quantifiable Data:

- *“How many families here have stopped selling products due to climate impacts in the past 2 years?”*

VII. Conflicts, Migration, and Demands

Technique: Post-its + Clustering (collective prioritization).

1. Key Questions:

- *“What conflicts have emerged or worsened because of climate change? (e.g., water, land, invasions).”*
- *“Has anyone migrated temporarily for climate-related reasons? To where and why?”*
- *“If you could demand three actions from the government, what would they be?”* (Write on post-its, group by theme, and vote).

VIII. Closing

1. Participatory Validation:
   - Summarize the group’s three main conclusions (facilitator writes on flipchart).
2. Acknowledgment and Next Steps:
   - *“How would you like these results to be used?”*
   - Offer to provide a summary report for the community.
