# Supplementary material for: Climate resilience through bioeconomy: A mixed-methods protocol for assessing adaptation policies in rural settlements on the Amazon
Source: PLoS One. 2026 Feb 13;21(2):e0342911. doi: 10.1371/journal.pone.0342911 (PMC12904387; doi:10.1371/journal.pone.0342911)
Supplement: S3 File — (PDF) [file pone.0342911.s003.pdf]

Interview Guide: Mapping Climate Policies and Bioeconomy Strategies in Rural Pará

I. Interviewee Profile and Municipal Context

(Objective: To characterize governance profiles and climate vulnerability for comparative analysis)

1. Name:
2. Position/Role:
3. Department/Agency:
4. Municipality:
5. Years in current position:
6. Years of experience in public service:

Basic Climate Governance Data:
7. Does the municipality have:

- A formal local climate adaptation plan? ( ) Yes ( ) No
- A climate change mitigation policy? ( ) Yes ( ) No
- If yes, is it aligned with national frameworks (PNMC) or global frameworks (NDC/Paris Agreement)?

II. Perceptions on Climate Change and Policy Integration

8. What are the most severe climate impacts observed in rural areas (e.g., agricultural losses, water scarcity, extreme events)?
   - These impacts are documented by:
     [ ] Meteorological data
     [ ] Agricultural loss reports
     [ ] Local traditional knowledge
9. Considering the overall challenges and responsibilities of the department, what level of importance is given to climate issues? (1 = not important / 5 = very important)
   - ( ) 1 ( ) 2 ( ) 3 ( ) 4 ( ) 5
10. How do municipal policies explicitly address climate adaptation/mitigation in rural livelihoods?

- Do they incorporate scientific thresholds (e.g., 1.5°C scenarios, IPCC risk assessments)?

11. Are there specific bioeconomy interventions (e.g., agroecology, community forest management, payment for environmental services)?

- How do they compare with references such as the EU Bioeconomy Strategy or the Amazon Concertation Pact?

III. Financing and Institutional Capacity

12. Budget transparency:

- What % of the municipal budget is allocated to climate actions? Is there a monitoring system (e.g., climate tagging)?
- Breakdown for the last 3 years:

13. Are there performance indicators for climate policies (e.g., GHG reduction, hectares under sustainable management)?

IV. Climate Impacts on Rural Systems

14. Documented climate effects in:

- **Agriculture:** Crop variability, pests, changes in crop suitability
- **Forestry:** Fires, availability of non-timber forest products, degradation rates
- **Water:** Conflicts among users, aquifer depletion, irrigation restrictions

15. Are traditional knowledge systems integrated into adaptation planning (e.g., fire management by Indigenous peoples, flood forecasting)?

V. Bioeconomy and Sustainable Transitions

16. Which bioeconomy value chains are prioritized?

- Non-timber forest products (e.g., açaí, Brazil nuts)
- Agroforestry systems
- Bioenergy/biofuels
- Ecotourism
- **Barriers to scaling up:** Certification costs? Market access?

17. Does the municipality encourage:

- Agroecological transition (e.g., subsidies for organic inputs)?
- Land restoration (e.g., compliance with CAR, low-carbon agriculture)?

VI. Multi-level Governance

18. How do rural communities participate in policy design?

- Mechanisms: [ ] Public hearings [ ] Councils [ ] Participatory budgeting

19. Are there partnerships with academia/CSOs (e.g., climate-smart agriculture pilots, satellite monitoring)?

VII. Policy Challenges and Gaps

20. Please rate the obstacles to implementation (scale 1–5, where 1 = minor obstacle and 5 = major obstacle):

- Lack of technical capacity
- Insufficient financing
- Political resistance
- Data gaps

21. Policy recommendations: Which interventions would improve rural climate resilience?

VIII. Final Validation

22. May we access supporting documents (e.g., climate plans, budget reports)?
23. Additional comments:
