# Supplementary material for: Climate resilience through bioeconomy: A mixed-methods protocol for assessing adaptation policies in rural settlements on the Amazon
Source: PLoS One. 2026 Feb 13;21(2):e0342911. doi: 10.1371/journal.pone.0342911 (PMC12904387; doi:10.1371/journal.pone.0342911)
Supplement: Attachment — Submitted filename: PONE-D-25-53058.pdf [file pone.0342911.s004.pdf]

PLOS ONE

Climate resilience through bioeconomy: A mixed-methods protocol for assessing adaptation policies in rural settlements on the amazon

--Manuscript Draft--

**Manuscript Number:**	PONE-D-25-53058
**Article Type:**	Study Protocol
**Full Title:**	Climate resilience through bioeconomy: A mixed-methods protocol for assessing adaptation policies in rural settlements on the amazon
**Short Title:**	A mixed-methods protocol for assessing adaptation policies in rural settlements on the amazon
**Corresponding Author:**	Daniel Silva, Ph.D.<br>Federal University of Southern and Southeastern Para: Universidade Federal do Sul e Sudeste do Para<br>Marabá, Para BRAZIL
**Keywords:**	
**Abstract:**	Introduction<br>Climate change intensifies social, economic, and environmental vulnerabilities in Amazonian rural communities, where dependence on natural resources and limited institutional capacity constrain adaptation. The bioeconomy is often proposed as an alternative pathway for sustainable development, yet empirical evidence of its role in resilience remains scarce.<br>Objective<br>This protocol outlines a mixed-methods study designed to evaluate public policies for climate adaptation and to analyze community resilience strategies in agrarian reform settlements in Pará, Brazil.<br>Methods<br>The study will be conducted in 10 municipalities across the three regional superintendencies of the National Institute for Colonization and Agrarian Reform. The design integrates (i) secondary data on socioeconomic, climatic, and policy indicators; (ii) semi-structured interviews with municipal managers (n = 1–3 per municipality); (iii) focus groups with community leaders (n = 8–12 per settlement); and (iv) household surveys (n = 20–50 families per settlement). Primary outcomes include the Income and Livelihood Diversification Index (ILDI), Climate Vulnerability Perception Scale (CVPS), Bioeconomy Engagement Index (BEI), and Reported Adaptive Capacity Index (RACI). Secondary measures include the Adaptation Strategy Diversity Index (ASDI) and a Municipal Climate Engagement Typology (MCET). Quantitative analyses will employ multilevel linear and generalized linear models (LMM/GLMM), while qualitative data will be analyzed through reflexive thematic analysis, with final integration achieved via a triangulation framework.<br>Expected results<br>The study will generate integrated indicators of vulnerability, adaptive capacity, and bioeconomy engagement, enabling the identification of spatial, socioeconomic, and institutional patterns associated with resilience. Findings are expected to inform municipal and state policymakers, strengthen local adaptation initiatives, and contribute to the achievement of Sustainable Development Goals (SDGs 2, 13, and 15).
**Order of Authors:**	Daniel Silva, Ph.D.
	Larissa Alves
	Naurinete Reis
	Maclem Erane
	Vitor Castro
	Emilio Mendes
**Additional Information:**	

Powered by Editorial Manager® and ProduXion Manager® from Aries Systems Corporation

Question	Response
**Financial Disclosure**<br><br>Enter a financial disclosure statement that describes the sources of funding for the work included in this submission. Review the submission guidelines for detailed requirements. View published research articles from *PLOS ONE* for specific examples.<br><br>This statement is required for submission and **will appear in the published article** if the submission is accepted. Please make sure it is accurate.<br><br>**Funded studies**<br>Enter a statement with the following details:<br>• Initials of the authors who received each award<br>• Grant numbers awarded to each author<br>• The full name of each funder<br>• URL of each funder website<br>• Did the sponsors or funders play any role in the study design, data collection and analysis, decision to publish, or preparation of the manuscript?<br><br>Did you receive funding for this work?	Yes
Please add funding details.<br>as follow-up to "**Financial Disclosure**<br><br>Enter a financial disclosure statement that describes the sources of funding for the work included in this submission. Review the submission guidelines for detailed requirements. View published research articles from *PLOS ONE* for specific examples.<br><br>This statement is required for submission and **will appear in the published article** if the submission is accepted. Please make sure it is accurate.	This research was funded by the National Council for Scientific and Technological Development (CNPq), Brazil, through Call 19/2024 – Pro Amazônia, grant number 445058/2024-2 awarded to DS. The funder had no role in the design of the study; in the collection, analyses, or interpretation of data; in the writing of the manuscript; or in the decision to publish the results.

**Funded studies**<br>Enter a statement with the following details:<br>• Initials of the authors who received each award<br>• Grant numbers awarded to each author<br>• The full name of each funder<br>• URL of each funder website<br>• Did the sponsors or funders play any role in the study design, data collection and analysis, decision to publish, or preparation of the manuscript?<br><br>Did you receive funding for this work?"	
Please select the country of your main research funder (please select carefully as in some cases this is used in fee calculation).<br>as follow-up to "**Financial Disclosure**<br><br>Enter a financial disclosure statement that describes the sources of funding for the work included in this submission. Review the submission guidelines for detailed requirements. View published research articles from *PLOS ONE* for specific examples.<br><br>This statement is required for submission and **will appear in the published article** if the submission is accepted. Please make sure it is accurate.<br><br>**Funded studies**<br>Enter a statement with the following details:<br>• Initials of the authors who received each award<br>• Grant numbers awarded to each author<br>• The full name of each funder<br>• URL of each funder website<br>• Did the sponsors or funders play any role in the study design, data collection and analysis, decision to publish, or preparation of the manuscript?<br><br>Did you receive funding for this work?"	BRAZIL - BR
**Competing Interests**	The authors have declared that no competing interests exist.

Use the instructions below to enter a competing interest statement for this submission. On behalf of all authors, disclose any competing interests that could be perceived to bias this work—acknowledging all financial support and any other relevant financial or non-financial competing interests.<br><br>This statement is required for submission and **will appear in the published article** if the submission is accepted. Please make sure it is accurate and that any funding sources listed in your Funding Information later in the submission form are also declared in your Financial Disclosure statement.<br><br>View published research articles from *PLOS ONE* for specific examples.<br><br>**NO authors have competing interests**<br><br>Enter: *The authors have declared that no competing interests exist.*<br><br>**Authors with competing interests**<br><br>Enter competing interest details beginning with this statement:<br><br>*I have read the journal's policy and the authors of this manuscript have the following competing interests: [insert competing interests here]*<br><br>* typeset	
**Ethics Statement**<br><br>Enter an ethics statement for this submission. This statement is required if the study involved:<br><br>• Human participants<br>• Human specimens or tissue<br>• Vertebrate animals or cephalopods<br>• Vertebrate embryos or tissues	This study involves human participants and was reviewed and approved by the Research Ethics Committee (CEP) of the Federal University of Pará (UFPA), in accordance with Brazilian National Health Council Resolution No. 510/2016. The approval number is CAAE: 87492325.6.0000.0018.<br><br>Informed consent will be obtained from all participants prior to their inclusion in the study. For community members, the consent process will involve providing the Informed Consent Form (ICF) in writing, reading it aloud, and explaining it in clear and accessible language, considering possible variations in literacy levels; written consent will then be obtained by signature or fingerprint. For public managers, written consent

Powered by Editorial Manager® and ProduXion Manager® from Aries Systems Corporation

• Field research<br><br>Write "N/A" if the submission does not require an ethics statement.<br><br>General guidance is provided below. Consult the submission guidelines for detailed instructions. **Make sure that all information entered here is included in the Methods section of the manuscript.**	will be obtained.

Powered by Editorial Manager® and ProduXion Manager® from Aries Systems Corporation

**Format for specific study types**<br><br>**Human Subject Research (involving human participants and/or tissue)**<br>• Give the name of the institutional review board or ethics committee that approved the study<br>• Include the approval number and/or a statement indicating approval of this research<br>• Indicate the form of consent obtained (written/oral) or the reason that consent was not obtained (e.g. the data were analyzed anonymously)<br><br>**Animal Research (involving vertebrate animals, embryos or tissues)**<br>• Provide the name of the Institutional Animal Care and Use Committee (IACUC) or other relevant ethics board that reviewed the study protocol, and indicate whether they approved this research or granted a formal waiver of ethical approval<br>• Include an approval number if one was obtained<br>• If the study involved *non-human primates*, add *additional details* about animal welfare and steps taken to ameliorate suffering<br>• If anesthesia, euthanasia, or any kind of animal sacrifice is part of the study, include briefly which substances and/or methods were applied<br><br>**Field Research**<br><br>Include the following details if this study involves the collection of plant, animal, or other materials from a natural setting:<br>• Field permit number<br>• Name of the institution or relevant body that granted permission	
**Data Availability**<br><br>Authors are required to make the data underlying their research findings fully available, without restriction.<br><br>If the manuscript reports pilot or preliminary results, the data underlying those results must be made available at the time of publication. A Data Availability	N/A - No results are reported

Powered by Editorial Manager® and ProduXion Manager® from Aries Systems Corporation

Statement describing where the data can be found is required at submission. Your answers to this question constitute the Data Availability Statement and will be published in the article, if accepted. When legal or ethical requirements prohibit public data sharing, authors must indicate how researchers may obtain access to the data. See the PLOS Data Policy and FAQ for detailed information.<br><br>**Important:** Stating 'data available on request from the author' is not sufficient. If your data are only available upon request, select 'No' for the first question and explain your situation in the text box.<br><br>For protocols without pilot or preliminary data, authors are strongly encouraged to state how they plan to share research data from their study when it is completed or published.<br><br>If your study includes pilot data, will they be made fully available without restriction?	
**Describe where the data may be found in full sentences. If you are copying our sample text, replace any instances of XXX with the appropriate details.**<br><br>• If **no pilot data are reported** in the manuscript, please enter the following: *No datasets were generated or analysed during the current study. All relevant data from this study will be made available upon study completion.*<br>• If the manuscript reports pilot data **held in a public repository**, include URLs, accession numbers or DOIs. If this information will only be available after acceptance, indicate this by ticking the box below. For example: *All XXX files are available from the XXX database (accession number(s) XXX, XXX.).*<br>• If the manuscript reports pilot data contained **within the manuscript and/or**	No datasets were generated or analysed during the current study. All relevant data from this study will be made available upon study completion.

**Supporting Information files**, enter the following:<br>*All relevant data are within the manuscript and its Supporting Information files.*<br>• If neither of these applies but you are able to provide **details of access elsewhere**, with or without limitations, please do so. For example:<br><br>*Data cannot be shared publicly because of [XXX]. Data are available from the XXX Institutional Data Access / Ethics Committee (contact via XXX) for researchers who meet the criteria for access to confidential data.*<br><br>*The data underlying the results presented in the study are available from (include the name of the third party and contact information or URL).*<br>• This text is appropriate if the data are owned by a third party and authors do not have permission to share the data.<br><br>For study protocols with data management or sharing plans, authors are encouraged to briefly describe in their Data Availability Statement how data they generate will be made accessible when the study is completed. For example:<br>• Deidentified research data will be made publicly available when the study is completed and published.<br><br>* typeset	
Additional data availability information:	Tick here if the URLs/accession numbers/DOIs will be available only after acceptance of the manuscript for publication so that we can ensure their inclusion before publication.

Powered by Editorial Manager® and ProduXion Manager® from Aries Systems Corporation

Manuscrito

Click here to access/download;Manuscript;Research_Protocol_English.pdf

Climate resilience through bioeconomy: A mixed-methods protocol for assessing adaptation policies in rural settlements on the amazon

Daniel Silva [1¶], Larissa Alves [2&], Naurinete Reis [3&], Maclem Erane [3&], Emilio Mendes [4&], Vitor Castro [5&]

[1] Institute for Agrarian and Regional Development Studies, Federal University of Southern and Southeast Pará, Pará, Brazil;

[2] Institute of Economics, State University of Campinas, São Paulo, Brazil;

[3] Center for Affirmative Actions, Federal University of Southern and Southeast Pará, Pará, Brazil;

[4] Institute of Applied Social Sciences, Federal University of Pará, Pará, Brazil;

[5] Institute of Geosciences, Federal University of Southern and Southeast Pará, Pará, Brazil.

* Corresponding author
E-mail: daniel.nogueira@unifesspa.edu.br

[¶] These authors contributed equally to this work.

[&] These authors also contributed equally to this work.

Funding and Disclosure

Funded by National Council for Scientific and Technological Development (CNPq) through Call 19/2024 Pro Amazônia.

Competing Interests

The authors declare that there are no competing interests.

Data Availability Statement

This is a study protocol and does not contain original data. The data management plan and the data collection instrument (questionnaires, interview guides, and focus group guides) are provided as Supporting Information. Upon completion of the study, all anonymized data will be made publicly available in accordance with the plan proposed in the Data Management section.

Abstract

Introduction

Climate change intensifies social, economic, and environmental vulnerabilities in Amazonian rural communities, where dependence on natural resources and limited institutional capacity constrain adaptation. The bioeconomy is often proposed as an alternative pathway for sustainable development, yet empirical evidence of its role in resilience remains scarce.

Objective

This protocol outlines a mixed-methods study designed to evaluate public policies for climate adaptation and to analyze community resilience strategies in agrarian reform settlements in Pará, Brazil.

Methods

The study will be conducted in 10 municipalities across the three regional superintendencies of the National Institute for Colonization and Agrarian Reform. The design integrates (i) secondary data on socioeconomic, climatic, and policy indicators; (ii) semi-structured interviews with municipal managers (n = 1–3 per municipality); (iii) focus groups with community leaders (n = 8–12 per settlement); and (iv) household surveys (n = 20–50 families per settlement). Primary outcomes include the Income and Livelihood Diversification Index (ILDI), Climate Vulnerability Perception Scale (CVPS), Bioeconomy Engagement Index (BEI), and Reported Adaptive Capacity Index (RACI). Secondary measures include the Adaptation Strategy Diversity Index (ASDI) and a Municipal Climate Engagement Typology (MCET). Quantitative analyses will employ multilevel linear and generalized linear models (LMM/GLMM), while qualitative data will be analyzed through reflexive thematic analysis, with final integration achieved via a triangulation framework.

Expected results

The study will generate integrated indicators of vulnerability, adaptive capacity, and bioeconomy engagement, enabling the identification of spatial, socioeconomic, and institutional patterns associated with resilience. Findings are expected to inform

municipal and state policymakers, strengthen local adaptation initiatives, and contribute to the achievement of Sustainable Development Goals (SDGs 2, 13, and 15).

Introduction

Climate change represents one of the main threats to sustainable development, especially for vulnerable rural communities in regions of high ecological sensitivity such as the Amazon [1]. These populations face complex challenges in which poverty, limited access to public policies, and dependence on fragile production systems interact with environmental impacts, exacerbating their vulnerability [2, 3]. The intensification of extreme events, including prolonged droughts and atypical floods, has already compromised the livelihoods of communities dependent on family farming and natural resources [4].

In this context, the bioeconomy has emerged as a potential pathway to reconcile economic development and environmental conservation, relying on the sustainable use of biodiversity and the valorization of traditional knowledge [5]. However, its implementation faces critical challenges, including conceptual ambiguity - where differing definitions may or may not generate socio-environmental benefits [6] - and the pace of climate change, which may limit the feasibility of economic models dependent on ecosystems already under pressure [7].

Recent scholarship emphasizes that advancing the Amazonian bioeconomy requires not only technological innovations but also multi-scalar policy changes and institutional support [8]. From a sustainability transitions perspective, the effective scaling of socio-bioeconomy initiatives depends on improvements in infrastructure, value chains, and social

organizations, which remain unevenly developed across region [9]. Research on non-timber forest products (NTFPs) - such as açaí, Brazil nut, and babassu - demonstrates their essential role in linking biodiversity conservation with livelihood generation. Yet, while production, distribution, and economic aspects are relatively well documented, legal, regulatory, and socio-political frameworks remain underexplored [10]. Moreover, rural families' interactions with forests extend beyond economic considerations, encompassing cultural, subsistence, and spiritual dimensions, which complicates the straightforward promotion of bioeconomy value chains. Together, these insights highlight the need for a comprehensive, context-sensitive approach that integrates economic, ecological, and social perspectives.

Parallel to these debates, studies show that Amazonian communities are already devising local adaptation strategies to climate variability and extreme events. Research using a Community-Based Adaptation (CBA) lens has documented a wide range of coping, adaptive, and transformative responses to extreme floods, despite limited governmental support [11]. Other work shows how floodplain residents integrate natural hydrological and ecological processes into their management systems, optimizing ecosystem functioning through traditional knowledge [12]. Similarly, farmers in various regions have adopted new crop varieties, irrigation systems, and strategic land-use practices to enhance resilience. Yet they continue to face barriers such as water scarcity, high input costs, and insufficient technical assistance, underscoring the importance of institutional support and access to sustainable technologies [13].

These findings demonstrate the capacity of rural communities to devise locally grounded adaptation responses, but they also reveal persistent structural challenges, particularly

weak integration of local strategies into formal public policies. Importantly, while evidence on adaptation is expanding, a critical gap remains in understanding how these processes unfold in agrarian reform settlements, which represent one of the largest rural populations in the Amazon and sit at the intersection of climate vulnerability, land reform, and bioeconomy development.

Despite recognition of the importance of climate adaptation, there is still no standardized methodology to assess local impacts and adaptive responses in Amazonian rural contexts [14]. This limits the formulation of effective public policies, which are often based on generic indicators that fail to reflect the specific realities of vulnerable populations. Similarly, bioeconomy initiatives, although widely promoted, still lack robust evidence of their effectiveness in scenarios of accelerated climate change [15].

This protocol seeks to address these gaps by combining the assessment of climate adaptation and mitigation policies with the analysis of bioeconomy chains in agrarian reform settlements. To our knowledge, this represents an unprecedented perspective in the international literature on adaptation in tropical regions. The results will contribute not only to filling local data gaps but also to advancing global debates on climate adaptation, bioeconomy, and sustainable development, directly engaging with the Sustainable Development Goals (SDG 2 – Zero Hunger, SDG 13 – Climate Action, and SDG 15 – Life on Land).

Materials and Methods

Objectives, hypotheses, and research strategies

This study protocol was developed with the aim of identifying and analyzing public policies and community strategies for climate change mitigation and adaptation in municipalities and rural communities in the Eastern Brazilian Amazon. The specific research objectives are: (i) To map and quantify climate vulnerability through the construction and analysis of secondary indicators of exposure, sensitivity, and adaptive capacity. (ii) To identify, through field research, the perceived effects of climate change, adaptation and mitigation strategies, and the main barriers faced by family farmers in selected rural settlements. (iii) To map municipal and state-level public policies for addressing climate change, including an analysis of the public budget allocated to these actions. (iv) To analyze the perception, implementation challenges, and articulation of climate policies from the perspective of local public managers.

The approach integrates public policy indicators (e.g., existence of mitigation and adaptation plans, climate budget) with community perceptions, allowing for a broader analysis of ongoing impacts and adaptation strategies. The data collected under this protocol aims to test hypotheses based on (i) spatial patterns; (ii) socioeconomic patterns; (iii) demographic patterns; (iv) institutional/political patterns; and (v) bioeconomy/climate patterns.

Table 1 summarizes the main hypotheses guiding this work.

Table 1 – Hypotheses Guiding the Protocol

Analyzed Pattern	Primary Hypothesis (PH)	Theoretical Premise
**Spatial and Environmental**	PH1: Settlements with greater forest cover and ecological connectivity exhibit higher adaptive capacity, reflected in a greater	Intact ecosystems provide more ecosystem services (provisioning, regulating) that function as buffers

	diversity of bioeconomy practices and a lower perception of climate vulnerability.	against climate impacts and as a foundation for resilient economic strategies [16].
**Socioeconomic and Livelihoods**	PH2: Household income diversification (agricultural, extractive, non-agricultural) is positively associated with higher perceived climate resilience and greater adoption of adaptation actions.	Diversification reduces dependence on a single activity vulnerable to climate events, enabling the absorption of shocks and the reallocation of resources [17].
**Demographic and Social Capital**	PH3: Multigenerational demographic composition and participation in community organizations are positively correlated with the adoption of a more diversified portfolio of adaptation strategies (both traditional and innovative).	Intergenerationality facilitates the transmission of traditional knowledge, while social organization facilitates access to information, resources, and external policies [18].
**Institutional and Public Policies**	PH4: The existence and implementation of participatory public policies on climate and bioeconomy explicitly at the municipal level are associated with higher adaptive capacity reported by communities and greater productive diversification based on socio-biodiversity.	Well-designed and participatory policies provide the resources, incentives, and structures necessary for communities to implement effective adaptation strategies at scale [19].
**Bioeconomy and Climate**	PH5: Engagement in bioeconomy value chains (particularly processing and certification) is positively associated with higher household income levels and the adoption of climate mitigation and adaptation practices.	Value addition and access to differentiated markets create economic incentives for conservation and for investment in sustainable and resilient practices [20, 21].

Primary and Secondary Outcomes

Based on the hypotheses presented in Table 1, this protocol pre-specifies a set of primary and secondary outcomes to ensure analytical parsimony, clarity, and replicability.

Primary outcomes (core indicators of household resilience and bioeconomy engagement):

1. Income and Livelihood Diversification Index (ILDI): a continuous score (0–1) that synthesizes the distribution of agricultural, extractive, and non-agricultural income. *Expected direction*: higher values will be associated with greater adoption of adaptation actions (HP2).
2. Climate Vulnerability Perception Scale (CVPS): an average score (1–5) constructed from items measuring climate risk perception. *Expected direction*: lower perceived vulnerability in settlements with greater forest cover and ecological connectivity (HP1).
3. Bioeconomy Engagement Index (BEI): an ordinal variable (level 0: no engagement; 1: primary production; 2: processing; 3: processing + certification). *Expected direction*: greater engagement will be associated with higher average household income and adoption of mitigation practices (HP5).
4. Reported Adaptive Capacity Index (RACI): a reflective score derived from farmers' self-assessment of their ability to respond to climate events, used to test HP4.

Secondary outcomes (exploratory measures to enrich analysis):

a) Adaptation Strategy Diversity Index (ASDI): a count of reported practices, used to test HP3.

b) Municipal Climate Engagement Typology: a contextual variable (high, medium, or

low engagement) constructed from the analysis of municipal plans and public budgets (HP4).

All outcomes will be operationalized on continuous, ordinal, or categorical scales, as appropriate, and incorporated into multilevel models (LMM/GLMM), with families (level 1) nested within settlements and municipalities (level 2).

Sample, Inclusion and Exclusion Criteria, and Participant Characteristics

The study will be conducted in the state of Pará, in the Eastern Brazilian Amazon. The selection of this state as the study area is strategically grounded in its centrality to debates on agrarian development, environmental conservation, and climate justice in the Brazilian Amazon [22]. Critically, Pará concentrates approximately 25% of all rural settlements in the country, hosting about 27% of all settled families in Brazil, representing the largest absolute population in agrarian reform projects within a single state. This demographic density of settlements, located in the Amazon biome - which is globally recognized as one of the most climate-sensitive - creates a unique and crucial socioecological context for investigation [23].

Focusing on this state allows the examination, on a microscale, of the challenges and adaptation strategies of a vast and vulnerable population, whose experiences are directly shaped by the intersection of land tenure policies, deforestation pressures, and the intensification of climate extremes. Therefore, the results generated from this sample will not only have significant local relevance but will also provide transferable insights to

other regions of the Pan-Amazon facing similar dynamics, directly contributing to filling evidence gaps in climate adaptation and mitigation policies grounded in social equity.

To operationalize research in this unique context, the study will be conducted in ten municipalities of Pará, strategically distributed across the three regional superintendencies of the National Institute for Colonization and Agrarian Reform (INCRA).

The choice of 10 municipalities is not arbitrary but rather designed to capture the diverse socioecological contexts of agrarian reform settlements in Pará. These municipalities encompass distinct territorial and economic features that are central to understanding adaptation dynamics in the Amazon. They include: (i) areas strongly influenced by mining activities, where extractive industries create both opportunities and vulnerabilities; (ii) regions dominated by agribusiness expansion, with intensive land-use change and pressure on family farming; (iii) floodplain and dryland environments, which present contrasting climate risks and livelihood strategies; (iv) municipalities intersected by major highways such as the Transamazon Highway, where infrastructure development shapes access to markets and exposure to environmental impacts; and (v) extractive and conservation-oriented settlements, where socio-biodiversity chains and traditional livelihoods play a central role.

By sampling across this range of contexts, the study ensures that the results reflect not only average conditions but also the structural heterogeneity of Amazonian rural settlements, thereby enhancing both the analytical robustness and the transferability of findings to other regions facing similar challenges.

In each selected municipality, up to two agrarian reform settlements will be sampled, ensuring representation of the main settlement modalities nationally recognized: Settlement Project (PA), Agroextractive Settlement Project (PAE), Extractive Reserve (RESEX), Sustainable Development Project (PDS), and Collective Settlement Project (PAC). This approach will enable a robust comparative analysis of how different institutional designs and production bases mediate community vulnerability and adaptive capacity in the face of climate change.

Selection of settlements

Eligible settlements must have: (i) between 20 and 500 settled families, ensuring comparability and logistical feasibility; (ii) reflect production and socioeconomic conditions representative of the municipality; and (iii) be in a stage of creation, installation, structuring, or consolidation (0 to 7, according to INCRA's classification). Settlements with very particular or atypical conditions, such as experimental areas or arrangements that are not replicable, will be excluded.

Selection of families

In each selected settlement, up to 50 families will participate in the study, chosen through simple random sampling from official lists provided by local associations or INCRA. The inclusion criteria for families are: (i) residing in the settlement; (ii) having an adult household member available to respond to the questionnaire; and (iii) providing voluntary informed consent (ICF) (S1 File – Informed Consent Form). Families will be

excluded if: (i) they do not meet the residency requirement; (ii) they refuse or cannot provide consent; or (iii) communication barriers make the interview unfeasible.

Participant characteristics

Participants include: (i) local public managers (1–3 per municipality), responsible for municipal policies on agriculture, environment, or rural development; (ii) community leaders (8–12 per settlement), invited to participate in focus groups, representing different social and productive segments; and (iii) settled families (up to 50 per settlement), covering a diversity of socioeconomic, productive, and demographic profiles. The study expects to include family farmers across different age groups, education levels, family compositions, and modes of engagement in local markets, as well as varying degrees of dependence on natural resources and participation in public policies. This sampling strategy seeks to balance territorial representativeness, social diversity, and logistical feasibility, enabling comparisons across municipalities, settlement types, and family profiles.

Qualitative saturation criterion

Qualitative data collection will follow the principle of theoretical saturation [24], assessed iteratively by the analysis team during coding. As a guiding strategy, we will consider saturation reached when two or more consecutive interviews or focus groups no longer generate new relevant codes. However, given the high heterogeneity of Amazonian settlements (e.g., floodplains, mining zones, forest-based communities), this criterion will be applied flexibly. Data collection may be extended in specific contexts if

new themes continue to emerge or if settlement-specific dynamics require further exploration. To ensure robustness, saturation will be evaluated both within settlement types (PA, PAE, RESEX, PDS, PAC) and across municipalities and will be triangulated with survey findings and field observations to confirm completeness.

Sample justification and statistical power

The study was designed to include 10 municipalities, up to 20 settlements, and 600 - 1,000 families. Power calculations were conducted using the simr package (version 4.1.1) in R. Simulations assumed a two-level structure (families nested within settlements) and were run for a range of intraclass correlation coefficients (ICCs: 0.05, 0.10, 0.15) to reflect plausible clustering effects based on prior rural surveys [25] and our pilot data. The simulations modeled a continuous outcome (e.g., ILDI) with a single binary predictor at the family level (e.g., participation in a bioeconomy chain). For a sample of 600 families across 20 settlements (average cluster size of 30), a significance level of $\alpha = 0.05$, and an ICC of 0.10, the design provides 82% power to detect a standardized mean difference (Cohen's d) of 0.3. The full simulation code will be included in the project's OSF repository upon study completion to ensure computational reproducibility.

Description of All Processes, Interventions, and Comparisons

Data collection will be carried out sequentially in two integrated phases, as detailed below. The methodological design was refined following a pilot study conducted in the

municipality of Marabá (PA), with the resulting adjustments described to ensure process transparency.

Phase 1: Secondary Data and Political-Institutional Context Analysis

This phase will focus on building a comprehensive diagnosis of the municipal context. It will involve, first, Climate Risk Assessment, through the compilation and analysis of secondary climate indicators widely recognized in the literature (e.g., temperature, precipitation, extreme events) from sources such as the National Institute of Meteorology (INMET) and the National Institute for Space Research (INPE), to characterize the exposure and sensitivity of the sampled municipalities. Second, Public Policy Mapping, through the identification and documentary analysis of municipal and state-level public policies related to climate change, agriculture, and rural development. This will include the analysis of master plans, laws, and decrees, with attention to both actions explicitly labeled as "climate-related" and sectoral actions with indirect impacts on adaptation and mitigation (e.g., sanitation, civil defense, family farming) [19]. Third, Public Budget Audit, applying a data mining methodology to municipal transparency portals [26, 27], which allows categorization and quantification of public expenditures directly or indirectly related to the climate agenda, thus overcoming the limitation of expenditures formally tagged as "climatic" [28].

Phase 2: Primary Data Collection with Key Stakeholders

This phase will capture the perceptions, experiences, and strategies of local actors through three instruments: (i) Household Surveys: administration of a structured

questionnaire to a sample of families in each settlement, collecting socioeconomic, productive, climate perception, and adaptation strategy data (S2 File – Household Survey); (ii) Focus Groups with Community Leaders: group discussions to obtain a consolidated view of vulnerabilities, conflicts, and collective adaptation strategies in the settlements, as well as to validate contextual information (S3 File – Focus Group Guide); (iii) Semi-Structured Interviews with Public Managers: interviews with municipal technicians and secretaries to understand perceptions, implementation challenges, and the articulation of climate policies from the perspective of local government (S4 File – Interview Guide).

Post-Pilot Methodological Adjustments.

Testing the instruments in Marabá (PA) resulted in crucial refinements. For Phase 1, the policy and budget analysis methodology was expanded to systematically capture implicit climate actions, as the pilot revealed that most municipal climate responses are driven by sectoral policies (e.g., agriculture, infrastructure) not explicitly labeled as climate related. For Phase 2, the household survey was adapted to include specific modules for riverine and floodplain communities, capturing particular climate impacts (e.g., changes in flood cycles, river-based access). The interview guide with managers was revised to replace technical jargon with more accessible terms, reflecting the fact that many municipalities lack a consolidated formal framework for climate policy.

Comparative Analysis Strategy

The analysis will be structured across four comparative levels, allowing the isolation of factors associated with resilience at different scales: (i) Across municipalities, contrasting indicators of vulnerability, climate budget, and policy robustness; (ii) Across settlement types (PA, PAE, RESEX, PDS, PAC), examining how different land tenure and institutional arrangements mediate convergencesoss families within settlements, correlating socioeconomic profiles (education, income diversification) with adoption of adaptation practices; (iv) Across social actors, triangulating the perspectives of public managers and communities to identify convergences and divergences in risk perceptions and policy effectiveness.

This multilevel approach is essential for identifying robust spatial, socioeconomic, and institutional patterns associated with climate resilience.

Data Analysis Plan

Data analysis will follow a sequential explanatory mixed-methods strategy (QUAN → QUAL) [29], in which the initial quantitative analysis will provide an overall picture to be subsequently deepened and contextualized by qualitative investigation. Triangulation of data from different sources (quantitative, qualitative, and secondary) will be employed to validate and enrich the findings, ensuring a robust understanding of the factors that influence climate resilience [30]. All analyses will be performed using R software (version 4.3.0 or higher) for the quantitative component and NVivo software (version 14 or higher) to support the organization and coding of qualitative data.

General Strategy and Integration

The analysis will occur in three main stages, corresponding to the data sets, culminating in an integration phase: (i) Secondary Data and Municipal Context Analysis, to characterize the macro context. (ii) Quantitative Primary Data Analysis (Survey), aimed at identifying patterns and testing hypotheses at household and community levels. (iii) Qualitative Primary Data Analysis (Interviews and Focus Groups), to explore causal mechanisms, perceptions, and nuances. (iv) Finally, integration and triangulation, with a synthesis of the findings to answer the research objectives.

Secondary Data and Municipal Context Analysis

The analysis of secondary data will proceed in three integrated steps to construct key municipal-level variables. First, a Municipal Climate Vulnerability Index (MCVI) will be created by compiling secondary indicators of exposure, sensitivity, and adaptive capacity from sources including AdaptaBrasil, MCTI, IBGE, and INPE. These indicators will be harmonized by converting to per capita measures where appropriate, standardized (z-scores), and aggregated through Principal Component Analysis (PCA) [31]. Second, municipal climate change plans will be assessed using categorical content analysis to generate a plan quality score. Third, a public expenditure audit, employing text mining of municipal transparency portals, will be conducted to identify and quantify climate-related budgets.

To ensure robustness, a multi-step strategy will address data quality challenges. For the MCVI, discrepancies or missing values will be handled using imputation techniques, with sensitivity analyses conducted to assess the impact on the index. Furthermore, all secondary data - including the budget analysis and plan documentation - will be cross-

checked against municipal transparency portals and triangulated with qualitative data from interviews and focus groups to ensure contextual accuracy.

Finally, the plan quality scores, and budget allocations will be combined to create a Municipal Climate Engagement Typology (e.g., High, Medium, Low). Both the MCVI and this engagement typology will be incorporated as contextual variables in the multilevel models.

Quantitative Primary Data Analysis (Survey)

Initially, descriptive statistics (frequencies, means, medians, and standard deviations) will be used to characterize the sample profile. Subsequently, to operate key concepts, composite indices will be constructed, and their internal consistency will be assessed using Cronbach's alpha ($\alpha > 0.7$) and/or Confirmatory Factor Analysis (CFA) [32]. The planned indices include Income and Livelihood Diversification Index (ILDI); Climate Adaptation Practices Index (CAPI); Bioeconomy Engagement Index (BEI); and Climate Vulnerability Perception Scale (CVPS).

To test the hypotheses in Table 1, linear mixed-effects models (LMM) and generalized linear mixed-effects models (GLMM) [33] will be applied, which are appropriate for hierarchical data (families nested within settlements and municipalities). The MCVI and the municipal engagement typology will be included as higher-level variables (level 2), while family characteristics (level 1) will be used as predictors and control variables.

Operationalization of the Indices

To ensure transparency and replicability, each index will be constructed according to clear theoretical and statistical criteria, as follows:

- Income and Livelihood Diversification Index (ILDI): formative index calculated using the Herfindahl-Hirschman formula $(1 - \sum pi^2)$ based on the relative shares of agricultural, extractive, and non-agricultural income. It will not be evaluated by Cronbach's alpha but described statistically.
- Climate Adaptation Practices Index (CAPI): reflective index, constructed from the standardized sum of reported adaptation practices (e.g., irrigation, resistant varieties, collective work). Internal consistency will be assessed by Cronbach's alpha ($\alpha \geq 0.7$) and, when appropriate, by confirmatory factor analysis.
- Bioeconomy Engagement Index (BEI): formative ordinal variable, with increasing levels of engagement in bioeconomy value chains (0: no participation; 1: primary production; 2: processing; 3: processing + certification/market differentiation).
- Climate Vulnerability Perception Scale (CVPS): reflective scale, constructed as the average of Likert-type items (1–5) capturing perceptions of climate risk (drought, flood, crop loss, food insecurity). Internal consistency will be assessed by Cronbach's alpha ($\alpha \geq 0.7$) and unidimensionality by CFA.
- Municipal Climate Vulnerability Index (MCVI): formative index based on indicators of exposure (climate), sensitivity (e.g., agricultural dependence, poverty), and adaptive capacity (e.g., education, policy access). It will be constructed from standardized indicators (z-scores) and aggregated through PCA.

PCA adequacy indicators ($KMO > 0.6$) and variance explained by the first component will be reported.

Each index will be statistically validated, described in terms of distribution, central tendency, and dispersion, and interpreted according to its reflective or formative nature.

Qualitative Primary Data Analysis

Transcriptions of interviews and focus groups will be analyzed using Braun and Clarke's Reflexive Thematic Analysis [34] in six stages: (1) familiarization; (2) generation of initial codes; (3) search for themes; (4) review of themes; (5) definition and naming of themes; and (6) report production. This analysis will identify central themes related to local perceptions, barriers, facilitators, and understandings of climate change, adaptation, and bioeconomy.

Integration and Triangulation

In the final phase, the findings will be integrated through a triangulation matrix [30]. This matrix will systematically map convergences, complementarities, and divergences between quantitative, qualitative, and secondary results for each research objective and hypothesis. For example, a quantitative pattern of low adoption of a bioeconomy practice (QUAN) will be examined considering reported barriers in interviews (QUAL) and the (non)existence of supportive policies identified in secondary data. This synthesis will enable not only the identification of *what* occurs but also *why* and *how* adaptation and resilience processes unfold.

Handling of Missing Data

The strategy for handling missing data will be determined by its mechanism and extent. During data collection, digital forms with mandatory field checks will minimize inadvertent missingness. Interviewers will be trained to probe for reluctant or "don't know" responses. Prior to analysis, we will generate a missingness pattern report. Little's Missing Completely at Random (MCAR) test will be used to inform the choice of handling method.

Our primary analysis will use a Full Information Maximum Likelihood (FIML) estimation within the mixed-effects models (lme4 package in R). FIML uses all available data points without discarding partial cases and provides unbiased estimates under the Missing at Random (MAR) assumption. To assess the robustness of our inferences to the MAR assumption, we will conduct sensitivity analyses using multiple imputation (mice package in R). We will create 20 imputed datasets, including all model variables and auxiliary variables correlated with missingness. The multilevel structure will be respected during imputation using the 2lonly method. Results from the pooled imputed models will be compared with the primary FIML analysis.

The amount and pattern of missing data for all key variables will be reported in the final publications.

Data Management Plan

A comprehensive data management plan has been developed to ensure the integrity, reproducibility, and controlled access of the data, while guaranteeing strict compliance with ethical protocols. All secondary data from public domains will be meticulously

documented and cited. Primary qualitative data (audio recordings and transcripts) and survey data, due to their sensitive nature and the risk of re-identification in communities with extensive sampling, will undergo a rigorous advanced anonymization process. This process includes the generalization of indirect identifiers and the suppression of excessively specific details.

Anonymized survey data, along with processing and analysis scripts and full documentation (metadata, data dictionary), will be deposited in the Open Science Framework (OSF) repository under a 12-month embargo from the date of publication of the primary results. After this period, the data will be made openly available. During the embargo, access requests can be submitted via the OSF platform or by emailing the corresponding author. Requests will be reviewed by the steering committee and granted upon approval of a brief research proposal and the signing of a data use agreement that prohibits re-identification attempts.

Raw qualitative data (audio and identifiable transcripts) will remain permanently confidential and restricted exclusively to the research team, as approved by the Ethics Committee. This framework seeks to balance the principles of scientific transparency and reproducibility with the fundamental protection of research participants' privacy and confidentiality.

Ethical Considerations

This study protocol was approved by the Research Ethics Committee (CEP) of the Federal University of Pará (UFPA), in accordance with Resolution No. 510/2016 of the

National Health Council, under CAAE number 87492325.6.0000.0018 (S5 File – REC Approval).

The Informed Consent Form (ICF) will be obtained from all participants prior to their inclusion in the study (S1 File – Informed Consent Form). For community members, the ICF will be provided in writing, reading aloud, and explained in clear and accessible language, considering possible variations in literacy levels. For public managers, the ICF will be presented in written form. All participants will be informed about the research objectives, the procedures involved, potential risks and benefits, the voluntary nature of participation, and their right to withdraw consent at any time without penalty. Written consent will be obtained by signature or fingerprint.

To ensure confidentiality, all data collected will be anonymized. Personal identifiers (names, specific locations, positions) will be removed and replaced with codes during transcription and data analysis. The digital database will be stored on a password-protected computer, and physical documents will be kept in a locked cabinet with restricted access to the research team. Results will be presented in aggregate form, ensuring that no individual participant can be identified.

Study Status and Timeline

The project is structured into three main phases, distributed over 36 months (2025–2027). As of the submission date of this protocol, the status of the study is as follows: ethical approval has been obtained, and a pilot study was conducted in the municipality of Marabá (PA) exclusively to refine the data collection instruments. Participant recruitment for this pilot occurred from September 08 to 26, 2025, and all participants provided

written informed consent. Large-scale data collection, as detailed in this protocol, has not yet started and is scheduled to run from November 03, 2025 to December 20, 2026.

One of the key factors contributing to the success of the pilot study in Marabá (PA) was the strong support and trust of local communities. This was made possible because several members of the research team were themselves selected from these communities, ensuring cultural familiarity, credibility, and direct involvement in fieldwork. In addition, the study has established formal and informal partnerships with local organizations, including universities, social movements, and civil society associations, which provide logistical support, facilitate dialogue with community leaders, and strengthen the ethical dimension of the research. These arrangements are not limited to Marabá (PA); rather, they extend to the other municipalities included in the study, where team members already have long-standing connections. This embedded approach enhances both the feasibility of data collection and the legitimacy of the research process, while reinforcing the principle of co-production of knowledge with local stakeholders.

The detailed timeline of activities is presented in Table 2 below.

Table 2 – Development and Implementation of the Study Protocol (2025–2026)

Etapas	Year 1				Year 2				Year 3			
	1T	2T	3T	4T	1T	2T	3T	4T	1T	2T	3T	4T
Preparation	X	X	X	X								
Instruments	X	X										
Recruitiment			X	X								
Training			X	X								
Data Collection (Pilot)			X									
Adjustment			X									

Field Research				X	X	X	X	X	X			
Formation of Local Teams				X	X							
Data Collection				X	X	X	X	X				
Data	X	X	X	X	X	X	X	X	X			
Secondary Data Survey	X	X										
Primary Data Curation			X	X	X	X	X	X	X			
Data Analysis	X	X	X	X	X	X	X	X	X			
Dissemination										X	X	X
Technical Reports										X		
Academic Articles										X		
Workshops										X	X	
Policy Brief										X		X

To ensure feasibility across a large and heterogeneous territory, the project relies on research teams established in each of the 10 selected municipalities. These teams are composed of local researchers and students, many of whom are members of the communities themselves, which facilitates trust and logistical access. The study is fully supported by funding from CNPq (Call 19/2024 – Pro Amazônia), which provides resources for fieldwork, data collection, and training. In addition, the protocol is strengthened by formal partnerships with local universities, social movements, and civil society organizations, which contribute infrastructure, mobilization, and institutional legitimacy. This decentralized structure, combined with strong local support, substantially reduces the logistical risks of conducting fieldwork in remote Amazonian areas and ensures the viability of the proposed design.

The preparation stage involves finalizing the data collection instruments based on insights gained from the pilot study, training the research team in ethical and methodological procedures, and establishing partnerships and logistical arrangements to ensure access to the study sites. Field research entails the systematic collection of secondary data, including municipal indicators, documentary evidence, and budget analyses, conducted in parallel with the collection of primary field data through household surveys, focus groups, and semi-structured interviews in the ten selected municipalities. Data analysis includes the curation, organization, and cleaning of datasets, followed by the statistical analysis of quantitative data, thematic analysis of qualitative data, and the integration and triangulation of findings using a mixed-methods framework. Dissemination comprises the preparation of manuscripts for submission to peer-reviewed scientific journals, the development of technical reports and policy briefs tailored for public managers, and the production of specific materials designed for local policymakers and community stakeholders. In addition, workshops will be organized to return and discuss the study's findings with the participating communities, ensuring that the results inform both academic debates and practical decision-making at the local level.

Discussion

Study Limitations

The main methodological advancement of this protocol lies in the integration of climate resilience indicators with bioeconomy dynamics in agrarian reform settlements. To the best of our knowledge, this approach has not been systematically applied in international studies, which underscores the novelty and global relevance of this work. However, some

limitations are anticipated. First, the reliance on secondary data is subject to the inherent limitations of public databases, such as variations in quality, completeness, and frequency of updates across municipalities. We will seek to mitigate this limitation through triangulation of data sources whenever possible.

Second, the qualitative component, based on interviews, may be subject to social desirability bias, in which participants provide responses they believe are expected by researchers rather than their genuine perceptions. To minimize this risk, interviewers will be trained to build rapport and foster a comfortable environment, assuring participants that there are no right or wrong answers.

Finally, the intentional selection of municipalities and settlements may limit the generalizability (external validity) of the results to the entire state of Pará. The study aims to provide an in-depth analysis of specific contexts, rather than a statistically representative overview. Nevertheless, the detailed methodological description will allow an assessment of the transferability of the findings to similar contexts.

Dissemination Plan

The results of this research will be disseminated through multiple channels to reach academic audiences, public policy managers, and the participating communities themselves.

For the academic audience, the primary results will be submitted for publication in international open-access, peer-reviewed journals (such as *PLOS Climate*, *Climate Policy*, or *Sustainability*) as well as in national journals. The findings will also be

presented at national and international scientific conferences in the fields of climate change, public policy, and Amazonian studies.

Dissemination to public managers and policymakers is a central point of this research and will be carried out through the preparation, in Portuguese, of a policy brief (executive summary) and a technical summary report in non-technical language specific to each municipality. These documents will be distributed to key actors, including the Pará State Secretariat for the Environment and Sustainability (SEMAS-PA), the State Secretariat of Agriculture (SAGRI-PA), and the municipal governments involved in the study. The goal is to directly inform the design and implementation of more effective climate policies.

Finally, in the participating communities, the results will be returned to the settlements through community workshops and the production of educational booklets. These workshops will be conducted in an accessible format, fostering dialogue about the results and discussing potential community adaptation strategies. This action is crucial to honor participants' contributions and ensure that the research generates local benefits.

Protocol Amendments and Early Termination

Any substantial modification to the study protocol - including changes in methodology, sample size, or participant eligibility criteria - will be submitted for approval by the Research Ethics Committee (CEP) prior to implementation. All changes will be documented in a master project file.

The study may be interrupted before its planned completion for the following reasons: (a) identification of unforeseen serious risks to participants or researchers; (b) insurmountable practical difficulties in data collection (e.g., inability to access key

communities or obtain public data); (c) formal withdrawal of consent by partner communities or local authorities.

If fieldwork constraints arise, we will reduce the sample to 6 municipalities while ensuring representation of settlement types.

In the event of early termination, the Principal Investigator will formally notify the Ethics Committee and the funding agency (CNPq). All data collected up to the point of termination will be anonymized, securely stored, and may be used for analysis, with the circumstances of the interruption clearly disclosed in any resulting publication.

Author’s Contributions

Conceptualization: Daniel Silva.

Data curation: Vitor Castro.

Formal analysis: Larissa Alves, Emilio Mendes.

Funding acquisition: Daniel Silva.

Investigation: Larissa Alves, Naurinete Reis, Maclem Erane, Emílio Mendes.

Methodology: Daniel Silva, Larissa Alves, Naurinete Reis, Maclem Erane, Emilio Mendes.

Project administration: Daniel Silva.

Software: Vitor Castro

Supervision: Daniel Silva.

Validation: Naurinete Reis.

Visualization: Emilio Mendes.

Writing – original draft: Daniel Silva.

Writing – review and editing: Daniel Silva, Larissa Alves, Naurinete Reis, Maclem Erane, Emilio Mendes, Vitor Castro.

Acknowledgments

The authors would like to express their sincere gratitude to the rural communities and public managers of the state of Pará who, with their availability and insights, were fundamental to the data collection for this study. We also thank the National Council for Scientific and Technological Development (CNPq) for the financial support that made this research possible through Call 19/2024 Pro Amazônia.

Supporting Information

S1 File. Methodology for the analysis of budget data

S2 File. Focal Groups Guide

S3 File. Interview Guide for Public Managers

S4 File. Questionnaire Guide for Family Farmers

S5 File. Informed Consent Form (ICF) used

S6 File. Research Ethics Committee (REC) approval – the official document certifying project approval

References

1. Flores BM, Montoya E, Sakschewski B, et al (2024) Critical transitions in the Amazon forest system. Nature 626:555–564

2. Bangalore M, Hallegatte S, Bonzanigo L, Kane T, Fay M, Narloch U, Treguer D, Rozenberg J, Vogt-Schilb A (2016) Shock Waves: Managing the Impacts of Climate Change on Poverty. https://doi.org/10.1596/978-1-4648-0673-5

3. Rana IA, Khan MM, Lodhi RH, Altaf S, Nawaz A, Najam FA (2023) Multidimensional poverty vis-à-vis climate change vulnerability: Empirical evidence from flood-prone rural communities of Charsadda and Nowshera districts in Pakistan. World Development Sustainability 2:100064

4. Calvin K, Dasgupta D, Krinner G, et al (2023) IPCC, 2023: Climate Change 2023: Synthesis Report. Contribution of Working Groups I, II and III to the Sixth Assessment Report of the Intergovernmental Panel on Climate Change [Core Writing Team, H. Lee and J. Romero (eds.)]. IPCC, Geneva, Switzerland. https://doi.org/10.59327/IPCC/AR6-9789291691647

5. Ferraz D, Pyka A (2023) Circular economy, bioeconomy, and sustainable development goals: a systematic literature review. Environmental Science and Pollution Research. https://doi.org/10.1007/s11356-023-29632-0

6. Vieira ICG, Fernandes DA, Araújo R, Freitas MAB, Brandão F (2024) Scaling up sociobioeconomy in the Amazon: Opportunities and risks. One Earth 7:1908–1912

7. Talwar N, Holden NM (2022) The limitations of bioeconomy LCA studies for understanding the transition to sustainable bioeconomy. International Journal of Life Cycle Assessment 27:680–703

8. Vieira ICG, Fernandes DA, Araújo R, Freitas MAB, Brandão F (2024) Scaling up sociobioeconomy in the Amazon: Opportunities and risks. One Earth 7:1908–1912

9. Garrett R, Ferreira J, Abramovay R, et al (2024) Transformative changes are needed to support socio-bioeconomies for people and ecosystems in the Amazon. Nat Ecol Evol 8:1815–1825

10. Araujo ECG, Silva TC, da Cunha Neto EM, Favarin JAS, da Silva Gomes JK, das Chagas KPT, Fiorelli EC, Sonsin AF, Maia E (2024) Bioeconomy in the Amazon: Lessons and gaps from thirty years of non-timber forest products research. J Environ Manage 370:122420

11. Almudi T, Sinclair AJ (2022) Community-level responses to extreme flooding: the case of Brazilian Amazon *ribeirinhos*. Clim Dev 14:781–793

12. Oviedo AFP, Mitraud S, McGrath DG, Bursztyn M (2016) Implementing climate variability adaptation at the community level in the Amazon floodplain. Environ Sci Policy 63:151–160

13. Monteverde C, Quandt A, Gilberto de Souza Ribeiro J, De Sales F (2024) Changing climates, changing lives: Voices of a Brazilian Amazon farming community in a time of climate crisis. PLOS Climate 3:e0000522

14. Reyes-García V, Álvarez-Fernández S, Benyei P, et al (2023) Local indicators of climate change impacts described by indigenous peoples and local communities: Study protocol. PLoS One. https://doi.org/10.1371/journal.pone.0279847

15. Kimura LT, Andrade ER, Nobre I, Nobre CA, de Medeiros BAS, Riaño-Pachón DM, Shiraishi FK, Carvalho TCMB, Simplicio MA (2023) Amazon Biobank: a collaborative genetic database for bioeconomy development. Funct Integr Genomics 23:101

16. Xiong J, Yu H, Li L, Yuan M, Yu J (2025) Asymmetry between ecosystem health and ecological quality from an Earth observation perspective. Sci Rep. https://doi.org/10.1038/s41598-025-94717-4

17. Said Z, Vigneshwaran P, Shaik S, Rauf A, Ahmad Z (2025) Climate and carbon policy pathways for sustainable food systems. Environmental and Sustainability Indicators. https://doi.org/10.1016/j.indic.2025.100730

18. Lissillour R, Essiz O, Boninsegni MF, Song Z (2025) Intergenerational transmission of sustainable consumption practices: Dyadic dynamics of green receptivity, subjective knowledge, peer conformity, and intra-family communication. J Environ Manage 378:124754

19. Rogers NJL, Adams VM, Byrne JA (2023) Factors affecting the mainstreaming of climate change adaptation in municipal policy and practice: a systematic review. Climate Policy 23:1327–1344

20. Piñeiro V, Arias J, Dürr J, et al (2020) A scoping review on incentives for adoption of sustainable agricultural practices and their outcomes. Nat Sustain 3:809–820

21. Olagunju KO, Angioloni S, Canavari M (2025) Niche markets for sustainable agri-food systems: A systematic review. Heliyon. https://doi.org/10.1016/j.heliyon.2025.e42346

22. Franco MA, Rizzo L V., Teixeira MJ, et al (2025) How climate change and deforestation interact in the transformation of the Amazon rainforest. Nat Commun 16:7944

23. Costa F de A (2025) Rationale of the land market in the brazilian amazon and supply-demand oriented policies: A model and scenarios applied to Pará. Land use policy 158:107718

24. Rahimi S, khatooni M (2024) Saturation in qualitative research: An evolutionary concept analysis. Int J Nurs Stud Adv 6:100174

25. Dwivedi LK, Mahaptra B, Bansal A, Gupta J, Singh A, Roy TK (2023) Intra-cluster correlations in socio-demographic variables and their implications: An analysis based on large-scale surveys in India. SSM Popul Health 21:101317

26. Pizarro R, Delgado R, Eguino H, Pimenta C (2022) A Conceptual Framework for the Classification of Government Spending on Climate Change. https://doi.org/10.18235/0004449

27. Guzman S (2022) A Guide to Analyzing the Public Budget for Climate Action: A Citizen's Proposal. Founder of the Climate Finance Group for Latin America, and the Caribbean

28. Susskind L, Kim A (2022) Building local capacity to adapt to climate change. Climate Policy 22:593–606

29. Creswell J, Clark V (2018) Designing and Conducting Mixed Methods Research, 3rd ed. SAGE, Thousands Oaks

30. Fetters MD, Curry LA, Creswell JW (2013) Achieving Integration in Mixed Methods Designs—Principles and Practices. Health Serv Res 48:2134–2156

31. Gewers FL, Ferreira GR, Arruda HF De, Silva FN, Comin CH, Amancio DR, Costa LDF (2022) Principal Component Analysis. ACM Comput Surv 54:1–34

32. Hair Jr J, Black W, Babin B, Anderson R (2019) Multivariate Data Analysis, 8th ed. Cengage, Hampshire

33. Gelman A, Hill J (2006) Data Analysis Using Regression and Multilevel/Hierarchical Models. https://doi.org/10.1017/CBO9780511790942

34. Braun V, Clarke V (2006) Using thematic analysis in psychology. Qual Res Psychol 3:77–101

Click here to access/download
Supporting Information - Compressed/ZIP File Archive
Suplementary.zip
