# Supplementary material for: Climate resilience through bioeconomy: A mixed-methods protocol for assessing adaptation policies in rural settlements on the Amazon
Source: PLoS One. 2026 Feb 13;21(2):e0342911. doi: 10.1371/journal.pone.0342911 (PMC12904387; doi:10.1371/journal.pone.0342911)
Supplement: Attachment — Submitted filename: Response to Reviewers.pdf [file pone.0342911.s005.pdf]

REVIEWER #1

We would like to thank you for the careful reading of our manuscript and for the constructive and insightful comments. We appreciate the positive evaluation regarding the clarity, relevance, and overall structure of the study. Following your guidance, we have revised the manuscript extensively. The changes have substantially improved methodological transparency, theoretical grounding, clarity of objectives, and the international relevance of the protocol.

Below we provide a detailed, point-by-point response to all comments. All revisions have been incorporated in the updated manuscript.

1. Need for more citations in the Methods section

Reviewer comment:

"There is a lack of citations along the methods section... especially regarding the calculation of indexes and approaches for missing data analysis..."

Response:
We agree and have added multiple methodological references throughout the Methods section, including citations related to diversification measures, reflective and formative index construction, PCA, mixed-effects modeling, multiple imputation, and multilevel missing data handling. These additions strengthen transparency and replicability.

2. Need to summarize / merge certain sections (Data management, Ethics, Timeline)

Reviewer comment:

"Some sections in the methods could be significantly summarised and merged... data management, ethical considerations, project timeline."

Response:
We have condensed these sections substantially:

- Ethical Considerations was moved to the Methods section and shortened.
- Data Management was reduced to a concise paragraph describing anonymization, storage, and OSF deposition.
- Study Status and Timeline was reduced and summarized in a brief paragraph.

This improves the manuscript's focus on scientific content.

3. Discussion needs expansion (design rationale, contributions, international relevance)

Reviewer comment:

"The discussion needs to be significantly expanded… explain why the chosen study design is appropriate… highlight how the protocol builds upon previous studies… describe international relevance…"

Response:
The Discussion has been substantially expanded to include:

- A clear justification for the mixed-methods, multilevel design.
- An explanation of alternative designs (e.g., panel, quasi-experimental) and why they were not feasible.
- A comparison with existing literature showing how our protocol integrates climate adaptation, bioeconomy, governance, and resilience.
- A new section on the international relevance of the study and pathways for generalization.
- A description of how limitations will be accessed and communicated in the final results.

Sections that were not directly scientific (e.g., dissemination plan, early termination) were removed.

4. Need for a subsection describing qualitative data collection

Reviewer comment:

"There should be a subsection prior to 'Sample inclusion…' that describes them."

Response:
We added a new subsection titled "Data Collection Methods" before the sampling description, clarifying procedures for:

- household surveys
- semi-structured interviews
- focus groups

This ensures coherence with the qualitative saturation criteria.

5. Clarification of agrarian reform settlements and 'settled families'

Reviewer comment:

The reviewer did not understand these terms.

Response:
We added a clear definition in the Introduction and Sample section explaining that agrarian reform settlements are rural territories created through Brazil's federal land redistribution program (INCRA), and "settled families" refers to beneficiary households receiving land through this program. This addresses ambiguity for international readers.

6. Clarifications on indicator descriptions (range, direction, origin, aggregation)

Reviewer comment:

The reviewer requested more details for ILDI, CVPS, BEI, RACI, MCET.

Response:
We revised all indicators to include:

- range of values
- expected direction (associational)
- conceptual grounding
- whether original or adapted
- aggregation from household to settlement level (when applicable)

Descriptions are now consistent and transparent.

7. Avoiding overstatements (e.g., "no standardized methodology")

Reviewer comment:

The initial phrasing overstated the absence of methods.

Response:
We rewrote this section to present a more nuanced description, noting that existing methods are fragmented and unevenly applied, and clarifying that our protocol contributes by integrating climate adaptation, resilience, and bioeconomy in a multilevel framework.

Conclusion

We thank the reviewer and Academic Editor once again for the constructive feedback that greatly improved the clarity and contribution of the protocol. We respectfully resubmit the revised manuscript for further consideration.

REVIEWER #2

We thank you sincerely for your detailed and insightful review. Your comments significantly improved the methodological clarity, rigor, and overall contribution of our study protocol. We have carefully addressed every point raised and revised the manuscript accordingly. Below we provide a point-by-point response, following the structure of your comments.

1. Clarification of causal vs. associational language

Reviewer comment:

"The current framing occasionally suggests causal claims… Unless you add a credible identification strategy, the objectives and hypotheses should be clearly associational."

Response:

We agree and have revised the entire manuscript to remove any causal language. Specifically:

- All hypotheses (PH1–PH5) were rewritten using associational terms ("associated with," "co-occur with," "positively/negatively correlated with").
- We removed verbs implying causality ("leads to," "results in," "increases," "reflects"), replacing them with neutral correlational phrasing.
- The Objectives section was revised to explicitly state that the study aims to identify associations, not causal effects.
- We clarified the observational nature of the design in the Study Limitations.

These changes ensure full alignment with observational study and PLOS ONE guidance.

2. Pre-specification of confounders across levels & sensitivity analysis (Cinelli & Hazlett)

Reviewer comment:

"Pre-specify rich confounders… adopt Carlos Cinelli's framework for sensitivity analysis… robustness values and contour diagnostics."

Response:

We thank the reviewer for this excellent suggestion. We incorporated the following revisions:

- Added a new subsection explicitly listing confounders at the household, settlement, and municipal levels (market access, infrastructure, ecological

context, cooperatives, local associations, institutional capacity, political economy, and geographic factors).

- Added a detailed Sensitivity Analysis Plan, adopting the Cinelli & Hazlett [1] framework, with robustness values and contour plots to quantify the impact of unobserved confounding.
- Specified that analyses will be conducted using sensemakr, and results will report E-values/robustness thresholds.
- Clarified that this sensitivity analysis complements multilevel models and multiple imputation procedures.

These additions substantially strengthen the transparency and rigor of the analytical plan.

3. Expanded power calculations for multilevel design

Reviewer comment:

"Power deserves expansion to reflect clustering… ICC around 0.10… variation between communities… ordinal BEI harder to power… redo power and MDE calculations… grid of plausible ICCs… consider number of communities vs. number of respondents."

Response:

We fully revised the Power Calculations section to incorporate:

- A clear explanation of the role of ICCs and design effects.
- Simulations for continuous, binary, ordinal (CLMM), and count outcomes across ICCs = 0.05, 0.10, 0.20, 0.30.
- A statement that power is driven primarily by the number of settlements, not households.
- Justification for including 20–30 settlements in the sampling design.
- Minimum detectable effect estimates for:
    - LMM (continuous),
    - CLMM (BEI ordinal predictor/outcome),
    - and logistic models (binary outcomes).
- Discussion of trade-offs (increasing clusters > increasing respondents).

We revised the introductory sentence to emphasize that power calculations were used to determine the minimum sample size required, not merely to illustrate tool usage.

4. Explicit clarification of where variation is expected to come from

Reviewer comment:

"It would help to be explicit about where you expect variation to come from… most identifying variation will be between communities."

Response:

We added explicit statements in both the Sampling Strategy and Quantitative Analysis sections that:

- Most identifying variation is expected at the settlement (level-2) scale, given the importance of market integration, cooperative presence, mobility, and ecological context.
- This expectation guided our choice to maximize the number of settlements and informed the multilevel modeling approach.

This cross-referencing improves internal coherence.

5. Clarification of ordinal modeling for BEI

Reviewer comment:

"The move from a binary to a four-category engagement index is sensible… commit to an analysis approach appropriate for ordered categorical."

Response:

We revised the Analytical Plan to state explicitly:

- BEI will be modeled using cumulative link mixed models (CLMM) when used as an outcome.
- BEI may serve as a level-1 or level-2 predictor, with appropriate aggregation (means or distributions) at the settlement level when required.
- The proportional odds assumption will be checked.

This ensures appropriate treatment of the indicator.

6. Missing data plan, with clarity across levels

Reviewer comment:

"Please indicate how you will handle missingness at each level… multiple imputation respecting clustering… reweighting strategies… sensitivity."

Response:

We expanded the Missing Data section by:

- Differentiating missingness across household, settlement, and municipal levels.
- Specifying multilevel multiple imputation using mice and pan, with clustering preserved.
- Including inverse probability weighting (IPW) and augmented IPW for differential nonresponse.
- Adding procedures for secondary data missingness (climate indicators, municipal plans, budgets).
- Clarifying that missingness in municipal plans/budgets may be structural and analytically meaningful.
- Integrating this with the Cinelli-style sensitivity analysis.

This strengthens the rigor of the protocol.

7. Reduction of researcher degrees of freedom and primary outcomes

Reviewer comment:

"Pre-specifying a short list of primary outcomes and hypotheses… identify explicitly which variables will be used."

Response:

We created a new paragraph at the beginning of Primary Outcomes:

- Specifying exactly four primary outcomes (ILDI, CVPS, BEI, RACI).
- Listing which hypotheses (PH1–PH5) each primary outcome relates to.
- Clearly separating expected direction (conceptual meaning) from hypothesis testing (associational relationships).

Secondary outcomes are now explicitly labeled and described as complementary.

8. Qualitative standards (COREQ/SRQR) and mixed-methods integration (GRAMMS)

Reviewer comment:

"Commit to COREQ/SRQR… provide a GRAMMS-style description… triangulation table."

Response:

We added:

- A clear statement that qualitative reporting will follow COREQ.
- A full GRAMMS-consistent description of integration procedures.
- A triangulation table with decision rules for convergence, complementarity, and discordance.
- Updates to the Saturation and Qualitative Methods sections.

These revisions significantly clarify the role of qualitative data.

Conclusion

We sincerely appreciate the reviewer's contributions. The manuscript has improved substantially because of this detailed and thoughtful review. We believe the revised version now aligns fully with PLOS ONE's expectations for study protocols and provides a stronger, clearer, and more rigorous analytical framework.

We hope the revised manuscript meets with your approval, and we thank you again for your constructive feedback.

Sincerely,

Corresponding Author
