# Supplementary material for: Climate resilience through bioeconomy: A mixed-methods protocol for assessing adaptation policies in rural settlements on the Amazon
Source: PLoS One. 2026 Feb 13;21(2):e0342911. doi: 10.1371/journal.pone.0342911 (PMC12904387; doi:10.1371/journal.pone.0342911)
Supplement: Attachment — Submitted filename: Response to Reviewers (2.0).pdf [file pone.0342911.s007.pdf]

Response to Reviewer #1

Reviewer comment:

In the Discussion section, subsection “Study Limitations”:

“There are things in the text below that are not limitations. The discussion should not focus on limitations only. I would suggest removing any subheadings in this section and just organising the flow of topics logically.”

Response:

We thank the reviewer for this constructive suggestion. We agree that the original subsection extended beyond a discussion of limitations alone. In response, we removed the subheading and reorganized the text into a single, cohesive discussion that integrates methodological considerations, limitations, and study contributions in a more logical flow. This revision improves clarity and better reflects the purpose of the Discussion section. The revised text can be found in the Discussion section (lines 664 - 727).

In addition, statements identified by the reviewer as requiring further support are now appropriately referenced in the revised manuscript.

Response to Reviewer #2

Reviewer comment:

“You state that power calculations were performed with simulations for various ICCs and minimum detectable sizes, but you only present one of these simulations. It would be very helpful if you could add a compact table that shows these simulations…”

Response:

We thank the reviewer for this helpful suggestion, which we have incorporated into the revised manuscript. We now include a compact table reporting simulation-based minimum detectable effects (MDEs) for both continuous and ordinal outcomes across a range of intraclass correlation coefficients (ICC = 0.05, 0.10, 0.15, and 0.30). All simulations hold the total sample size fixed at N = 600 families, the number of clusters at K = 30 settlements, the significance level at $\alpha = 0.05$, and statistical power at 80%, as described in the Methods section.

This addition improves transparency by allowing readers to directly assess the sensitivity of the study design to different levels of clustering and to interpret potential null findings considering within-settlement correlation. The revised table appears in the Power Analysis subsection (lines 374 - 411).

To ensure computational transparency and reproducibility, the full simulation code has been made publicly available in the project's OSF repository.
